# Abnormal Detection in Big Data Video with an Improved Autoencoder

**DOI:** 10.1155/2021/9861533

**Published:** 2021-12-08

**Authors:** Yihan Bian, Xinchen Tang

**Affiliations:** ^1^School of Information Science and Technology, Shanghai Tech University, Shanghai 201210, China; ^2^Division of Science and Technology, Beijing Normal University-Hong Kong Baptist University United International College, Zhuhai 519087, China

## Abstract

With the rapid growth of video surveillance data, there is an increasing demand for big data automatic anomaly detection of large-scale video data. The detection methods using reconstruction errors based on deep autoencoders have been widely discussed. However, sometimes the autoencoder could reconstruct the anomaly well and lead to missing detections. In order to solve this problem, this paper uses a memory module to enhance the autoencoder, which is called the memory-augmented autoencoder (Memory AE) method. Given the input, Memory AE first obtains the code from the encoder and then uses it as a query to retrieve the most relevant memory items for reconstruction. In the training phase, the memory content is updated and encouraged to represent prototype elements of normal data. In the test phase, the learned memory elements are fixed, and reconstruction is obtained from several selected memory records of normal data. So, the reconstruction will tend to be close to normal samples. Therefore, the reconstruction of abnormal errors will be strengthened for abnormal detection. The experimental results on two public video anomaly detection datasets, i.e., Avenue dataset and ShanghaiTech dataset, prove the effectiveness of the proposed method.

## 1. Introduction

As a high-level computer vision task, video anomaly detection refers to the automatic detection of abnormal events in a given video sequence, which can effectively distinguish abnormal and normal activities and abnormal categories in the video. In the past few years, researchers have carried out many research related to anomaly detection [1–9]. Compared with normal events, events that rarely occur or have a low probability of occurrence are usually considered as abnormal ones. However, in practice, it is difficult to establish an effective anomaly detection model due to unknown event types and unclear definitions of anomalies. Most existing anomaly detection methods are designed based on the assumption that any pattern different from the learned normal pattern is regarded as anomalies. Based on such assumptions, the same activity in different scenarios may be represented as normal or abnormal events. For example, a fight scene where two people fight on the street may be considered abnormal, while the two people are normal when they are boxing. In addition, there is a large amount of redundant visual information in high-dimensional video data, which increase the difficulty of event representation in the video sequence.

According to the previous works, the anomaly detection methods can be generally divided into two types. Some anomaly detection methods are designed through reconstruction errors, which focus on modeling normal patterns in video sequences [3–5, 7, 8, 10, 11]. These methods learn the feature representation model of the normal pattern in the training phase and use the differences between the abnormal and normal samples to determine the final abnormal score of the test data during the testing phase, such as reconstruction errors or specific thresholds [7–14]. Although the reconstruction-based anomaly detection methods are good at reconstructing normal patterns in video sequences, the key problem with these methods is that they rely heavily on the training data. Another type of the method regards anomaly detection as a classification problem [15, 16]. For these methods, the anomaly score of a video sequence is predicted by using a trained classifier to extract features such as histogram of optical flow (HOF) or dynamic texture (DT). The performance of these methods is highly dependent on the training samples. In order to obtain satisfactory performance, extracting effective and discriminative features is essential for such anomaly detection methods [17–20]. However, the two types of methods usually model the interrelationships between events in a relatively simple way [7, 10, 21–23]. For example, only the linear relationship is considered, which is not enough for complex, highly nonlinear relationships in many real-world cases.

In recent years, methods based on deep learning were applied to the field of video detection and made great progress [24–26]. For example, the autoencoders (AE) use reconstruction errors to detect anomalies, and a series of methods have been improved on this basis. In addition, the generative adversarial networks (GAN) and long short-term memory (LSTM) have also been applied to solve the anomaly detection problem. However, the AE may have a strong generalization ability, resulting in the ability to reconstruct abnormal events. In [14], the researchers pointed out that because there are no abnormal training samples, the reconstruction of abnormal samples should be unpredictable, which may lead to larger reconstruction errors for abnormal samples. If some anomalies share a common composition pattern with normal training data or the decoder is “too strong” and cannot decode some anomaly codes well, then the AE can reconstruct the anomaly well.

In order to overcome the shortcomings of the AE, this paper uses a memory module to enhance the deep AE and develops a memory-augmented AE (Memory AE) method. When a new test sample is input, Memory AE will not directly encode it and input it into the decoder, but use it as a query to retrieve the relevant content in the memory module. Then, the content is aggregated and passed into the decoder. This process is realized by attention addressing. Furthermore, this paper uses differentiable shrinkage operators to induce the sparsity of memory addressing weights, which can encourage memory content to approach queries in the feature space. During the training phase, the encoder and decoder update the memory module at the same time to obtain a lower average reconstruction error. In the test phase, the learned memory content is fixed, and a small amount of normal memory items will be used for reconstruction. If these are selected as the neighborhood of the input code, the reconstruction error will be very obvious. Experiments on several public benchmark datasets show that the detection performance of Memory AE has reached the state of the art.

## 2. Principle of the Algorithm

**Figure 1:**
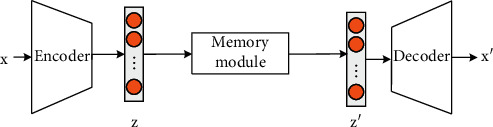
The flowchart of the proposed memory AE.

Figure 1 shows the overall network structure of Memory AE, which is divided into three substructures: encoder (used for encoding input and generating queries), decoder (used for reconstruction), and memory module (with memory and related addressing operations). As shown in Figure 1, given the event to be tested, the encoder first obtains its coded value. By using the coded value as a query, the memory module retrieves the most relevant content in the memory module through an attention-based addressing operator and then passes it to the decoder for reconstruction. During the training, the encoder and decoder optimize the parameters to minimize the reconstruction error and, at the same time, update the memory module to record the prototype elements of the encoded normal data. Given a test sample, the model uses only the limited normal patterns recorded in the memory module to perform reconstruction. In this way, the reconstruction tends to be close to the normal sample. Hence, the reconstruction error of the normal sample is small, and the abnormal error is large.

### 2.1. Encoders and Decoders

The encoder and the decoder are two parts of the AE. The former maps the input data to the feature space to obtain its coded value, and the latter reconstructs the coded value into the input data. The AE is composed of an encoder *f*_*w*_1__(·) and a decoder *g*_*w*_2__(·), which can be expressed as

(1) $$\begin{array}{c} z=f_{w_{1}}\left(x\right), \end{array}$$

(2) $$\begin{array}{c} x^{\prime }=g_{w_{2}}\left(z\right), \end{array}$$

where **x** and **x**′ are the input of the AE and the reconstructed input, respectively, **z** is the encoding results of **x**, and *W*_1_ and *W*_2_ denote the parameters of the encoder and the decoder, which can be obtained by minimizing the reconstruction error between **x** and **x**′:

(3) $$\begin{array}{c} \underset{W_{1},W_{2}}{\min}{\left\|x-x^{\prime }\right\|}_{2}^{2}. \end{array}$$

By reconstructing the error of the normal sample [19, 20] to determine whether it is abnormal, the AE has been successfully used to solve the abnormal detection task. However, the reconstruction of abnormal samples should be unpredictable, which may result in larger reconstruction errors for abnormal samples. In order to solve this problem, a memory module is introduced to the AE in Section 2.2, and a Memory AE is proposed.

### 2.2. Memory Module

The proposed method includes a memory module to record the prototype encoding mode and an addressing operation for accessing the memory module.

#### 2.2.1. Attention-Based Representation

The attention module is designed as a matrix *M* ∈ *ℝ*^*N*×*C*^ containing *N* real-time vector. For simplicity, assume that is *C* is the dimension of **z**; then, let Ζ=*ℝ*^*C*^. Given a row vector **m**_*i*_, ∀*i* ∈ [*N*], where [*N*] is an integer from 1 to *N*. Each represents *m*_*i*_ a memory item; given a set of queries **z** ∈ *ℝ*^*C*^, the memory network obtains $\hat{z}$ and replies with a soft address vector **w** ∈ *ℝ*^1×*N*^ as follows:

(4) $$\begin{array}{c} \overset{⌢}{z}=wM=\sum_{i=1}^{N}w_{i}m_{i}, \end{array}$$

where **w** is a row vector, and the sum of all items is 1, which represents *w*_*i*_, the item **w** of *i*. The weight vector can **w** be obtained by **z** calculation. As shown in equation (4), the address weight needs to be close **w** to the memory module. The mixed parameter is defined as *N*, the maximum capacity of the memory module. Although it is *N*, it is not easy to find the best for different datasets; fortunately, Memory AE is not sensitive to the setting of *N*. Sufficiently, large *N* can be well applied to each dataset.

#### 2.2.2. Attention for Memory Addressing

In Memory AE, the memory module is designed to record in detail the original normal mode *M* of the training phase. The memory module is defined as content addressable memory, $\overset{⌢}{\text{z}}$, and its addressing scheme calculates *w*, the attention weight, based on the similarity between the query and the memory item. As shown in Figure 1, each weight can be calculated through the softmax operation *w*_*i*_:

(5) $$\begin{array}{c} w_{i}=\frac{\exp\left(d\left(z,m_{i}\right)\right)}{\sum_{j=1}^{N}\exp\left(d\left(z,m_{j}\right)\right)}, \end{array}$$

where *d*(*·*, *·*) represents the similarity measure. This paper defines it as a cosine similarity:

(6) $$\begin{array}{c} d\left(z,m_{i}\right)=\frac{zm_{i}^{T}}{\left\|z\right\|\left\|m_{i}\right\|}. \end{array}$$

Just like equations (4)–(6), the memory module retrieves the most similar memory item to obtain a representation **z**. Due to the limitation of memory size and sparse addressing technology, only a small number of internal memory items can be addressed at a time. Therefore, the effective behavior of the memory module can be explained as follows. In the training phase, the decoder in Memory AE is limited to using very few addressable memory items for reconstruction, which requires effective use of memory items. Therefore, during the reconstruction, the memory module needs to be forced to record the most representative prototype mode in the input normal mode. In the test phase, given the trained memory, only the normal mode in the memory can be retrieved for reconstruction. Therefore, normal samples can be better reconstructed. Conversely, the coded value of the abnormal input can be replaced by the retrieved normal pattern, resulting in a large reconstruction error in the abnormal sample.

### 2.3. Training

Given a dataset containing samples {**x**^*i*^}_*i*=1_^*T*^, let **x**^*i*′^ denote the reconstruction samples of **x**^*i*^ in the training samples; the minimal refactoring is performed as follows:

(7) $$\begin{array}{c} R\left(x^{i}\text{,}x^{i{}^{\prime }}\right)={\left\|x^{i}-x^{i{}^{\prime }}\right\|}_{2}^{2}. \end{array}$$

The *l*_2_ norm is used to measure the reconstruction error; **w**^*i*′^ represents the memory addressing weight of each sample **x**^*i*^. In order to further promote the sparsity of **w**′, the sparse regularization is minimized during training. Considering that all **w**′ are nonnegative and ‖*w*′‖_1_=1, an optimization problem is formed as follows:

(8) $$\begin{array}{c} E\left(w^{i{}^{\prime }}\right)=\sum_{j=1}^{T}-w_{j}\cdot \;\log\left(w_{j}\right). \end{array}$$

By combining the loss function of equation (7) and equation (8), the objective function of Memory AE is as follows:

(9) $$\begin{array}{c} L\left(\theta_{e},\theta_{d},Μ\right)=\frac{1}{T}\sum_{i=1}^{T}R\left(x^{i},x^{i{}^{\prime }}\right)+\alpha E\left(w^{i{}^{\prime }}\right), \end{array}$$

where *α* is the hyperparameter in the training process. In practice, *α* is set to 0.0002. In the training process, the memory is updated through backpropagation and gradient descent. In backpropagation, only the gradient of the memory item with nonzero addressing weight can be nonzero.

### 2.4. Test

After the model is trained, the reconstruction error of the pixel at the position (*x*, *y*) of the *t*th frame *t* can be calculated by the following equation:

(10) $$\begin{array}{c} e\left(x,y,t\right)={\left\|I\left(x,y,t\right)-h\left(I\left(x,y,t\right)\right)\right\|}_{2}, \end{array}$$

where *h*(·) represents the entire model. Given the pixel-level reconstruction error of the *t*th frame, the reconstruction error of the entire frame of image can be obtained by summing *e*(*t*)=∑_(*x*, *y*)_*e*(*x*, *y*, *t*). Then, the anomaly score of the frame can be calculated as follows:

(11) $$\begin{array}{c} s\left(t\right)=1-\frac{e\left(t\right)-\min_{t}e\left(t\right)}{\max_{t}e\left(t\right)}. \end{array}$$

Finally, a threshold can be set to determine whether it is abnormal as *s*(*t*) > *θ*.

## 3. Experiment

In this section, the effectiveness of the proposed method is verified and compared with other existing methods. At present, two public datasets are used for experiments, i.e., the Avenue dataset and ShanghaiTech dataset. The frame-level area under the curve (AUC) and EER are used as quantitative evaluation indicators.

### 3.1. Preparation

The Avenue dataset uses a fixed camera with a resolution of 640 × 360 pixels to capture and record the street activities of the City University of Hong Kong. The dataset includes 16 training video clips containing normal human behavior and 21 test video clips containing abnormal events and human behavior. It has a total of 30652 frames, and all test videos have target-level annotations, that is, a rectangular area is used to mark anomalies in spatial locations. Normal behavior is people walking on the sidewalk, while abnormal events are people littering/discarding items, wandering, walking towards the camera, walking on the grass, and discarding objects.

The ShanghaiTech dataset is a very challenging collection for abnormal event detection. Unlike other datasets, it contains 13 different scenes with different lighting conditions and camera angles, including a total of 330 training videos and 107 test videos. The test set contains a total of 130 abnormal events with pixel-level annotations. The entire dataset has a total of 316154 frames, including 274515 frames in the training set, 42883 frames in the test set, and 17090 frames in the abnormal frame. The resolution of each video frame is 480 × 856.

The model is tested on a platform with NVIDIA GTX1080TI hardware platform and 8 GB video memory, and the software environment is PyTorch and Python 3.6. In order to measure the effectiveness of the method for video anomaly detection proposed in this paper, the AUC of the frame-level receiver operating characteristic curve (ROC) is used as the evaluation index. For frame-level evaluation indicators, if at least one pixel of a frame is marked as abnormal, the frame is considered abnormal. And the frame-level AUC is calculated by comparing the frame-level detection result with the frame-level of the real label.

### 3.2. Experimental Setup

All video frames are adjusted to 227 × 227 and then converted to grayscale images. The input of the model is 227 × 227 × 5, that is, 5 consecutive frames are used as the input of the model. After each convolutional layer, there is a batch normalization layer and a ReLU excitation layer. The decoder includes 4 deconvolution layers. The attention module is set to let each memory segment record a feature on a pixel in the feature map, corresponding to a subregion of the video segment. Therefore, the memory module is a 1000 × 64 matrix. The Adam optimizer is selected for the optimization of the entire model parameters. The initial learning rate is 0.0001, and the number of iterations is 1000. The momentum parameter is *ρ*_1_=0.9 and *ρ*_2_=0.999, and the batch size is 128.

### 3.3. Experimental Results

In order to prove the effectiveness of the proposed method in video anomaly detection, this paper compares it with 12 different existing methods. Among them, MPPCA (hybrid of probabilistic principal component analyzer) + SF (social power) [17] and MDT (hybrid of dynamic texture) [18] are methods based on manual features; Conv-AE [8], 3D Conv [19], Stacked RNN [20] and ConvLSTM-AE [21], MemNormality [10], and ClusterAE [22] are all methods based on autoencoders; AbnormalGAN [7] and Pred + Recon [23] are based on generating the adversarial networks' method.

Table 1 shows the frame-level video anomaly detection results of various methods. It can be observed that, in the results of the two datasets, the method based on AE is usually better than the method based on handmade features, and higher frame-level AUCs are obtained. This is because handmade features are usually extracted based on other tasks, and therefore may be suboptimal. In the AE-based methods, ConvLSTM-AE is better than Conv-AE because the former can better capture time information. In addition, it can also be noted that methods based on GAN perform better than most baseline methods. Finally, the Memory AE method proposed in this paper achieves 85.7% frame-level AUC on the Avenue data set, which is 0.6% ahead of the best-performing Pred + Recon [23] method; while the method proposed in this paper achieved 75.3% frame-level AUC on the ShanghaiTech dataset, which is 2% ahead of other methods in frame-level AUC, and the effect is very obvious. This is mainly because the proposed method based on Memory AE uses memory fragments, which can reconstruct anomalies well and introduce some random errors. In addition, compared with the Avenue dataset, the ShanghaiTech dataset has achieved a higher frame-level AUC. This is mainly because the ShanghaiTech dataset contains multiple scenes and abnormal events that have not appeared in other datasets before, which is more complicated. In order to verify the detection results of a single scene on the ShanghaiTech dataset, a single scene video segment is used for training and testing. 83 segments (25%) are used for training and 34 segments (32%) are used for testing, achieving 86.3% frame-level AUC, which has reached a level similar to that in the Avenue dataset. In summary, the proposed Memory AE method can be flexibly applied to different types of data. Only by using reconstruction errors, the proposed method can obtain better results with the least specific knowledge.

In order to evaluate the performance of the predefined memory module in detecting abnormal video events, the next step is to change the size of the memory module and perform experiments on the Avenue dataset, and the frame-level AUC values are given in Table 2. It can be found that given a sufficiently large memory module size, the Memory AE method can produce the best results robustly. When the size of the memory module is greater than 1000, the impact on the detection result is small, but the use of a larger memory module size will result in a greater amount of calculation, so the memory module size is selected as 1000.

**Figure 2:**
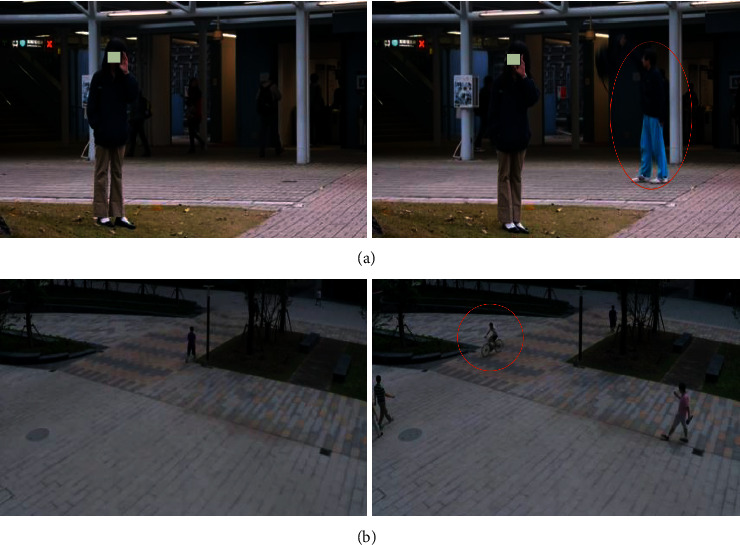
Examples of the detection results. (a) Example from the Avenue dataset. (b) Example from the ShanghaiTech dataset.

Figures 2(a) and 2(b), respectively, show some detection results in the Avenue dataset and ShanghaiTech dataset. The frames in the green box are normal frames from regular video clips, and the frames in the red box are abnormal frames from abnormal video clips. Some abnormal events such as dropping confetti, riding a bicycle on the sidewalk, beatings, etc., can be detected.

## 4. Conclusion

This paper proposes a Memory AE to improve the performance of big data anomaly detection in videos. Given input, the proposed Memory AE method first uses an encoder to obtain a coded representation and then uses the code as a query to retrieve the most relevant patterns in the memory module for reconstruction. Since the memory module is trained to record typical normal patterns, the proposed Memory AE can reconstruct normal samples well and enlarge the reconstruction error of abnormalities, which strengthens the role of reconstruction error as an abnormality detection standard. Experiments on two datasets prove the versatility and effectiveness of the proposed method. In the future, we will study the use of addressing weights for anomaly detection. Considering that the proposed memory module is universal and has nothing to do with the structure of the encoder and decoder, it will be integrated into a more complex basic model and used in experiments on more challenging datasets.

## Figures and Tables

**Table 1:** Comparison with the state-of-the-art methods in terms of AUC.

Method	Avenue	ShanghaiTech
MPPCA + SF	56.2%	—
MDT	77.4%	—
Conv-AE	80.0%	60.9%
Conv3D-AE	80.9%	—
Stacked RNN	81.7%	68.0%
ConvLSTM-AE	77.0%	—
MemNormality	88.5%	70.5%
ClusterAE	86.0%	73.3%
AbnormalGAN	—	72.4%
Pred + Recon	85.1%	73.0%
The proposed method	85.9%	75.4%

**Table 2:** The influence of the number of memory size on the experimental results of the Avenue dataset (frame-level AUC/%).

Size of the memory module	500	1000	1500	2000	2500
Result	78.3%	85.9%	85.4%	85.6%	85.8%

## Data Availability

The datasets used in this paper can be obtained from the corresponding author upon request.
